# Methylation Pattern Mediated by m^6^A Regulator and Tumor Microenvironment Invasion in Lung Adenocarcinoma

**DOI:** 10.1155/2022/2930310

**Published:** 2022-01-05

**Authors:** Feng Jiang, Yifang Hu, Xiaoqin Liu, Ming Wang, Chuyan Wu

**Affiliations:** ^1^Department of Neonatology, Obstetrics and Gynecology Hospital of Fudan University, Shanghai 200011, China; ^2^Department of Geriatrics, The First Affiliated Hospital of Nanjing Medical University, Nanjing 210029, China; ^3^Department of Pulmonary and Critical Care Medicine, The Affiliated Nanjing Drum Tower Hospital Clinical Medical College of Nanjing Medical University, Nanjing, 210008 Jiangsu, China; ^4^Plastic Surgery Department, The First Affiliated Hospital of Nanjing Medical University, Nanjing 210029, China; ^5^Department of Rehabilitation Medicine, The First Affiliated Hospital of Nanjing Medical University, Nanjing 210029, China

## Abstract

**Background:**

Recent research has established the existence of epigenetic modulation of the immune response. The possible involvement of RNA-n6-methyladenosine (m^6^A) alteration in tumor microenvironment (TME) cell invasion, on the other hand, is unknown.

**Methods:**

Based on 23 m^6^A regulators, we examined the alteration patterns of m^6^A in 629 LUAD tissues and comprehensively connected these modification patterns with TME cell invasion characteristics. The m^6^A score was calculated, and the m^6^A modification pattern of a single tumor was quantified using principal component analysis. Then, we further verified the expression of m^6^A related enzymes and the role hub gene (NOL10) closely related to survival in lung cancer cell lines.

**Results:**

Three separate m^6^A alteration modes have been discovered. TME cell invasion characteristics in the three modes were very similar to the three immunological phenotypes of tumors: immunological rejection, immunological inflammation, and immunological desert. We show that assessing the m^6^A modification pattern in a single tumor may help predict tumor inflammatory stage, subtype, TME interstitial activity, and prognosis. TME phenotypic inflammation is indicated by a high m^6^A score, which is characterized by elevated mutation load and immunological activation. The low m^6^A subtype showed matrix activation and ineffective immune infiltration, indicating that the TME phenotype of noninflammation and immunological rejection had a poor survival probability. Increased neoantigen burden was also linked to a high m^6^A score. Patients with a higher m^6^A score saw substantial therapeutic and clinical improvements. And reducing hub gene NOL10 expression substantially inhibited lung cancer cell growth and migration.

**Conclusions:**

This research shows that m^6^A alteration is critical in the creation of TME variety and complexity. The analysis of a single tumor's m^6^A alteration pattern will aid in improving our knowledge of TME invasion features and guiding more effective immunotherapy tactics.

## 1. Introduction

Lung cancer is the most frequent kind of cancer in the world, with the greatest fatality rate [1, 2]. Over the past decades, the incidence rate of lung adenocarcinoma, especially in women, has increased faster than that of squamous cell carcinoma [3]. According to data, adenocarcinoma has been the most prevalent kind of histology cancer in the globe since 2004 [4, 5]. But for the treatment of lung adenocarcinoma, the traditional treatment for patients is still limited. In recent years, immunotherapy appears and becomes popular because of its outstanding curative effect. PD1/PDL1 immune checkpoint inhibitors have been developed and used in the treatment of lung adenocarcinoma [6, 7]. However, only about 20% of patients benefit from immune checkpoint inhibitors [6]. A flurry of studies has revealed that the microenvironment in which cancer cells develop and survive is critical to tumor progression and treatment [8]. The ability of the tumor microenvironment (TME) to produce favorable and unfavorable effects on tumor development is different. Infiltrating immune cells can play an antitumor role; On the contrary, cancer cells can also culture stromal cells to promote cancer progression and metastasis [9]. TME has a crucial role in inpatient treatment response and prognosis, according to relevant tests conducted in recent years [10, 11]. The change of immune microenvironment may change the patients who did not respond to immunotherapy into patients who responded to immunotherapy [12]. How to screen these patients, or how to change the immune microenvironment in response to immunotherapy, is an important direction of immunotherapy research to improve the efficiency of immunotherapy.

With the further study of epigenetics in recent years, more than 100 kinds of RNA modifications have been known [13, 14]. Internal mRNA modification is mostly utilized to preserve mRNA stability [15]. The most common internal modifications of mRNA include N6 adenylate methylation (m^6^A), N1 adenylate methylation (m^1^A), and cytosine hydroxylation (m^5^C) [16]. Among the three types, the methylation of m^6^A is reversible, including the participation of methyltransferases (writers), demethylases (erasers), and methylated reading proteins (readers) [17]. m^6^A is first used to modify adenine (a) on RNA under the action of methyltransferase, and then, the RNA modified by m^6^A is demethylated under the action of demethylase during the process of RNA methylation. Finally, m^6^A modified RNA is recognized by methylated reading proteins and performs a series of downstream functions, including miRNA processing, mRNA translation, and splicing.

Methylation of m^6^A is involved in several biological activities, including tumor development and immunotherapy. However, current researches are limited to the impact of a single m^6^A regulator on tumor prognosis and immunotherapy [18, 19]. As a result, a thorough study of the invasive features of tumor microenvironment (TME) cells mediated by numerous m^6^A regulatory variables, as well as their implications for immunotherapy, would help us better understand how TMEs regulate their immune systems.

## 2. Methods

### 2.1. Data Source and Preprocessing of Lung Adenocarcinoma

**Figure 1:**
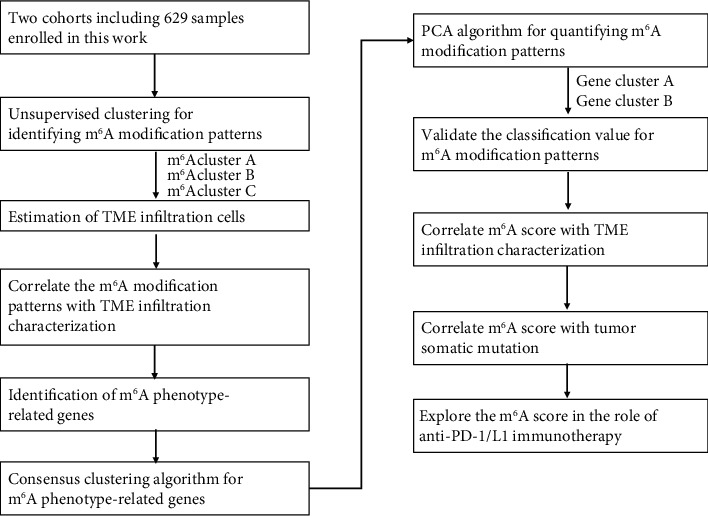
An overview of study design.

Figure 1 depicts the course of our research. Public gene-expression data and detailed clinical annotation were found in the Gene Expression Omnibus (GEO) and Cancer Genome Atlas (TCGA) databases. Patients who had no information about their prognosis were eliminated from the study. In this work, the LUAD cohorts (GSE26939) and TCGA-LUAD (the Cancer Genome Atlas-lung adenocarcinoma) were chosen for further investigation. GSE30219 and GSE37745 datasets were used for validation. The normalized matrix files were immediately downloaded for microarray data from the GEO database. For the TCGA datasets, RNA sequencing data (FPKM value) was obtained from the Genomic Data Commons (GDC, https://portal.gdc.cancer.gov/) [20]. The R package “limma” was then used to convert FPKM data to transcripts per kilobase million (TPM) numbers. The “ComBat” method of the “sva” package was used to correct batch effects caused by nonbiological technological biases. The TCGA database provided information on somatic mutations. The R (version 3.6.1) and R Bioconductor programs were used to examine the data.

### 2.2. Clustering of 23 m^6^A Regulators without Supervision

Researchers found unique m^6^A modification patterns regulated by m^6^A promoters by separating 23 regulators from each dataset. Among the 23 m^6^A regulators, there were eight writers (METTL3, METTL14, METTL16, WTAP, VIRMA, ZC3H13, RBM15, and RBM15B), two erasers (ALKBH5 and FTO), and thirteen readers (METTL3, METTL14, METTL16, WTAP, VIRMA, ZC3H13, RBM15, and RBM15B), two erasers (ALKBH5, YTHDC1, YTHDC2, YTHDF1, YTHDF2, YTHDF3, HNRNPC, FMR1, LRPPRC, HNRNPA2B1, IGFBP1, IGFBP2, IGFBP3, and RBMX). The expression of 23 m^6^A promoters was used to identify patients for future investigation using an unsupervised clustering approach. The grouping findings and their stability were analyzed using the consensus clustering technique [21]. The preceding stages were carried out using the ConsensuClusterPlus software, and 1000 times repeats were carried out to ensure classification stability [22]. Then, we do the survival analysis on the three clustering layers, using the “survival” and “survminer” R packets. For validation, we chose two more datasets (GSE31219 and GSE37745). These two datasets were combined, and the data was normalized. Three methylation-related patterns were discovered by unsupervised clustering of the gene expression of 23 m^6^A regulators in the sample. The three patterns' m^6^A regulator composition was nearly identical to that of the collected cohort. There have been many studies on m^6^A regulatory factors [18, 19, 23–26], so we selected three m^6^A regulatory factors (LRPPRC, RBMX, and METTL16) for further experimental verification.

### 2.3. Annotation of Functional Genes and Gene Set Variation Analysis (GSVA)

GSVA is an unsupervised, nonparametric method for detecting variance in route and biological process activity in expression dataset samples. To investigate the changes in biological processes between m^6^A alteration patterns, we utilized GSVA enrichment analysis and the “GSVA” R tools. The gene sets “c2.cp.kegg. v7.4. symbols” were obtained from MSigDB for GSVA analysis. A statistically significant *P* value was less than 0.05. The cluster profile R package was used to perform functional annotation for m^6^A-related genes, using a threshold value of 0.05.

### 2.4. TME Cell Infiltration Estimation

In the LUAD tumor microenvironment (TME), the ssGSEA (single-sample gene set enrichment analysis) methodology is employed to estimate the relative abundance of each cell infiltrate. The gene collection of immune cell types was obtained through previous related studies to mark each TME infiltration. The enrichment score derived by ssGSEA analysis indicates the relative abundance of each TME invading cell in each sample. Mariathasan et al. constructed a gene set to store genes related to some biological processes. We examined the link between the properties of m^6^A modification patterns and various linked biological pathways to learn more about them. Then, we used the ssGSEA method to assess the immunological features of each sample included in the research using 29 immune gene sets, which comprised genes linked to various immune cell types, functions, pathways, and checkpoints.

### 2.5. Differentially Expressed Genes (DEG) between Methylation-Related Patterns

To find m^6^A-related genes, we divided patients into three groups depending on their m^6^A modification patterns. Using the limma R package's empirical Bayesian technique, DEGs between different modification patterns were calculated [27]. The modified *P* value of 0.001 was used as the significant criterion for identifying DEGs.

### 2.6. Evaluation of the m^6^A Features and Generation of m^6^A score

To analyze the m^6^A modification pattern of a single lung adenocarcinoma patient—m^6^A gene features, which we call m^6^A score—we developed a scoring method to quantify the m^6^A modification pattern of a single tumor. The procedure for establishing m^6^A gene characteristics is as follows:

Before retrieving the overlap genes, the DEGs found in various m^6^A clusters were standardized across all datasets. For a subsequent study, the patients were divided into different groups and an unsupervised clustering technique was used to find overlap DEGs. The number of gene clusters and their stability was determined using the consensus clustering method. Then, using a multivariate Cox regression model, we did a prognostic assessment for each gene in the signature. For further investigation, the genes most related to prognosis were extracted. The m^6^A related gene signature was subsequently constructed using principal component analysis (PCA). Signature scores were chosen for both main component 1 and component 2. This technique had the advantage of focusing the score on the set with the largest block of highly correlated (or anticorrelated) genes, while downweighting the benefits of genes that did not track with other set components [28, 29]. Then, using a technique similar to GGI, we define the m^6^A score. m^6^A score = ∑(PC1*i* + PC2*i*), where *i* represents the expression of phenotypic-related genes for the m^6^A phenotype. We use the R language data package “limma,” “survival,” and “consensusclusterplu” to complete the above process. We analyzed the protein-protein interaction network (PPI) of 15 genes closely related to survival selected from 68 DEGs and then selected the hub gene with the most nodes for subsequent experimental verification.

### 2.7. Functional and Pathway Enrichment Study of Genes Associated with the m^6^A Phenotype

The expression of 23 m^6^A regulatory factors was then used to separate patients into three separate m^6^A modification types. The difference genes between various models were statistically analyzed using the “limma” package's empirical Bayes approach. The standard for determining the significance of the differential gene is the corrected *P* value < 0.001. We then performed GO analysis and KEGG analysis of by R data package. Using the R package “cluster profile” with a threshold of *P* value 0.05 and an adjusted *P* value of 0.05, Gene Ontology (GO) analysis was done to discover enriched GO keywords. The “gseKEGG” function from the R package “cluster profile” was used to determine the most connected pathways of overlapping genes.

### 2.8. Compilation of Immune-Related and Clinical Data

At https://tcia.at/patients, you may see the immune-related characteristics and genes of TCGA LUAD patients. Clinical information was gathered from the GEO dataset metadata and the TCGA database.

### 2.9. Cell Culture and Transfection

BEAS-2B, a human normal lung epithelial cell, and two lung cancer cell lines (H596 and A549) were grown at 37°C in a 5% CO_2_ incubator in Dulbecco's modified Eagle's medium (DMEM) with 10% foetal bovine serum (FBS). For subsequent experiments, the cells in the logarithmic phase were chosen. NOL10 knockdown was accomplished by utilizing Lipofectamine® 3000 transfection reagent (Invitrogen, USA) to transfect cells with NOL10-siRNA, as indicated by the manufacturer. 5′-GCUGCGGAGAAUAAUGUUUTT-3′ and 5′-UUUAAAUCGAUCAUCGGUGAG-3′ were the target sequences for siNOL10-1 and siNOL10-2.

### 2.10. RNA Extraction and Real-Time Quantitative PCR

Trizol reagent (Invitrogen, America) was used to extract total RNA from cell lines, and the HiScript Synthesis Kit was used to create cDNAs (Vazyme, China). Then, using the StepOnePlus Real-Time PCR system (Applied Biosystems, CA, USA) and the Fast SYBR Green Master Mix, a real-time quantitative PCR (qRT-PCR) analysis was carried out (Roche, America). The following are some of the primers: NOL10: forward-5′-CTGATGCTGCGGAGAATAATGT-3′, reverse-5′-ACTCTACCCTATGGTTCCTGT-3′.

### 2.11. Cell Counting Kit-8 (CCK-8) and Colony Formation

The proliferative potential of the cells was assessed using CCK-8 and plate colony formation tests. Cells were seeded at 2000 cells per well in 96-well plates overnight for the CCK-8 experiment, and cell growth was measured at various time points using the CCK-8 (C0038, Beyotime, China) according to the manufacturer's procedure. Enzyme labeling was used to measure the absorbance at 450 nm (Thermo Scientific Multiskan FC, USA). Each well of a six-well plate was filled with 1,000 cells from different groups for the colony formation experiment. When colonies could be seen, crystal violet and 4% paraformaldehyde were used to stain and fix the cells.

### 2.12. Transwell Invasion Assay

Invasion experiments were carried out in 24-well transwell chambers (Corning, USA) precoated with Matrigel (BD Biosciences, USA). In serum-free DMEM media, 2104 cells were planted into the top chambers of the transwell, and DMEM with 10% FBS was introduced to the lower chamber. The penetrated cells were preserved with 4 percent methanol and stained with 0.1 percent crystal violet after a 24-hour incubation period. An inverted microscope was used to picture and count each well at random (Nikon, Japan).

### 2.13. Western Blotting Analysis

RIPA lysis buffer was used to extract cell protein (P0013D, Beyotime, China). On 7.5 percent or 10% SDS-PAGE gels, equal quantities of protein samples were separated, and then electrotransferred onto nitrocellulose (NC) membranes (Pall Corporation, USA). The membranes were blocked for 2 hours at room temperature with 5% nonfat milk, then incubated overnight at 4°C with primary antibodies against RBMX (CST, 1 : 1,000), LRPPRC (Proteintech, 1 : 1,000), and METTL16 (Proteintech, 1 : 1,000), followed by 2 hours with the corresponding secondary antibody. Bands of conjugate proteins were observed using a Tanon 5200 multigel system after being washed three times more (Tanon Shanghai, China).

### 2.14. Statistical Analysis

The correlation coefficients between TME invading immune cells and m^6^A regulator expression were calculated using Spearman and distance correlation analysis. To compare two normally distributed groups, unpaired *t*-tests were utilized, while the Wilcoxon rank-sum test was used to examine nonnormally distributed data. One-way ANOVA and Kruskal-Wallis tests were used to assess differences between three or more groups [30]. To calculate the appropriate cutoff values for each cohort, the “surv-cutpoint” function in the “survminer” package was used. The Kaplan-Meier method was used to generate survival curves for the prognostic analysis, and log-rank tests were employed to compare groups. The univariate Cox regression model was used to compute the hazard ratios (HRs) for m^6^A regulators and m^6^A phenotype-related genes. *P* values were always two-sided, and *P* < 0.05 was considered statistically significant. All statistical analyses were performed using R 3.6.1 (https://www.r-project.org/).

## 3. Results

### 3.1. LUAD m^6^A Regulating Factor Genetic Variant

**Figure 2:**
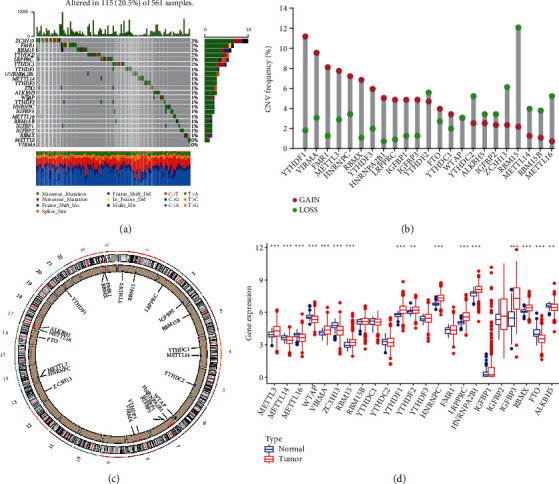
(a) The mutation frequency of 23 m^6^A regulators in 561 LUAD patients from the TCGA cohort. Each column represented individual patients. The upper barplot showed TMB; the number on the right indicated the mutation frequency in each regulator. The right barplot showed the proportion of each variant type. The stacked barplot below showed a fraction of conversions in each sample. (b) The CNV variation frequency of m^6^A regulators. The height of the column represented the alteration frequency. The deletion frequency, blue dot; the amplification frequency, red dot. (c) The location of CNV alteration of m^6^A regulators on 23 chromosomes. (d) The expression of 23 m^6^A regulators between normal tissues and LUAD tissues. Tumor, red; normal, blue. The upper and lower ends of the boxes represented the interquartile range of values. The lines in the boxes represented median value, and black dots showed outliers. The asterisks represented the statistical *P* value (^∗^*P* < 0.05; ^∗∗^*P* < 0.01; ^∗∗∗^*P* < 0.001).

In LUAD, we looked at the frequency of copy number variation and somatic mutations in 23 m^6^A regulatory components. Among the 561 samples from the TCGA database, 115 samples had a mutation of the m^6^A regulator, with a frequency of 20.5% (Figure 2(a)). According to the findings, ZC3H13 exhibited the greatest mutation frequency, followed by FMR1, RBM15, YTHDC2, LRPPRC, and YTHDC1, while VIRMA and METTL3 had no mutation in LUAD samples. When it came to copying number variation (CNV), YTHDF1, VIRMA, and FMR1 had a high rate of amplification, but RMB15 and METTL16 were mostly copy number losses (Figure 2(b)). We further mapped the position of the regulatory factors of m^6^A on the chromosome (Figure 2(c)). To examine whether the aforementioned genetic variation altered the expression of the m^6^A regulator in LUAD patients, we looked at the mRNA expression levels of regulatory factors in normal and LUAD samples. We discovered that CNV alteration may be the key cause for m^6^A regulatory factor expression to be disrupted. In LUAD tissues, the expression of m^6^A regulatory factors (such as IGFBP3 and HNRNPC) was considerably higher than in normal lung tissues, and vice versa (such as ZC3H13 and METTL16) (Figure 2(d)). We also looked at the protein levels of RBMX, METTL16, and LRPPRC in lung cancer cells and discovered that RBMX and LRPPRC were upregulated, whereas METTL16 was downregulated in lung cancer cells relative to normal lung epithelial cell BEAS-2B (Figure S1).

### 3.2. Methylation Patterns of m^6^A Mediated by 23 Regulators

**Figure 3:**
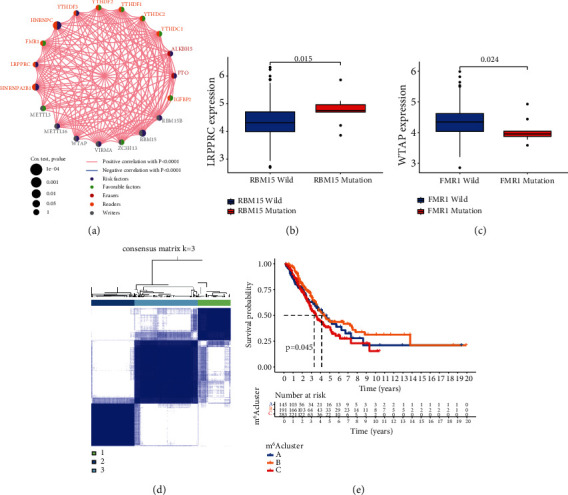
(a) The interaction between m^6^A regulators in LUAD. The circle size represented the effect of each regulator on the prognosis, and the range of values calculated by the log-rank test was *P* < 0.001, *P* < 0.01, *P* < 0.05, and *P* < 0.1, respectively. Purple dots, risk factors of prognosis; green dots, protective factors of prognosis. The lines linking regulators showed their interactions, and thickness showed the correlation strength between regulators. A negative correlation was marked with blue and a positive correlation with red. The regulator of erasers, readers, and writers was marked with red, yellow, and gray, respectively. (b) Difference in the LRPPRC expression between RBM15-mutant and RBM15 types. (c) Difference in the WTAP expression between FMR1-mutant and FMR1 types. (d) Consensus matrices of the cohort for *k* = 3. (e) Survival analyses for the three m^6^A modification patterns based on 629 patients with LUAD from two cohorts including 145 cases in m^6^A cluster A, 191 cases in m^6^A cluster B, and 283 cases in m^6^A cluster C. Kaplan-Meier curves with log-rank *P* value 0.045 showed a significant survival difference among three m^6^A modification patterns. The m^6^A cluster B showed significantly better overall survival than the other two m^6^A clusters.

In LUAD patients, a univariate Cox regression model demonstrated the predictive importance of 23 m^6^A regulators (Figure S2 and Table S1). We used the m^6^A network of regulatory factors to describe the interaction between regulatory factors, regulatory factor connections, and their prognostic significance (Figure 3(a)). We found that not only the expression of the same functional class of m^6^A regulatory factors was significantly correlated but also there was a significant correlation among the three regulatory factors. Considering the relatively high mutation frequency of writing genes ZC3H13, FMR1, and RBM15, we analyzed the difference of gene expression between mutant and wild type (Figures 3(b) and 3(c)). WTAP expression was dramatically reduced in FMR1 mutant tumors compared to wild-type tumors, whereas LRPPRC expression was dramatically increased in RBM15 mutant tumors. It shows the interaction between reader and writer, but the interaction is different according to the different regulatory factors. Because m^6^A methylation regulators may contribute to LUAD heterogeneity and are linked to the tumor microenvironment, unsupervised clustering was used to identify novel possible m^6^A methylation regulator patterns based on the expression of 23 m^6^A methylation regulators in the LUAD cohort. The grouping effect of the three clusters is the best, as illustrated in Figure S3. The 629 patients were divided into m^6^A methylation type A (147 cases), B (195 cases), and C (288 cases) (Figure 3(d)). The prognosis analysis of the three major subtypes of m^6^A showed that the survival rate of the m^6^A cluster B subtype was particularly high (Figure 3(e)). We chose the GSE30219 and GSE37745 datasets related to lung adenocarcinoma since they had the most extensive survival information to further evaluate the results of methylation patterns. Unsupervised clustering of the gene expression of 23 m^6^A regulators in the sample revealed three methylation-related trends (Figure S4A-D). The three patterns' m^6^A regulator composition was virtually identical to that of the assembled cohort (Figure S4E).

### 3.3. Infiltration Characteristics of TME Cells in Different m^6^A Modified Models

**Figure 4:**
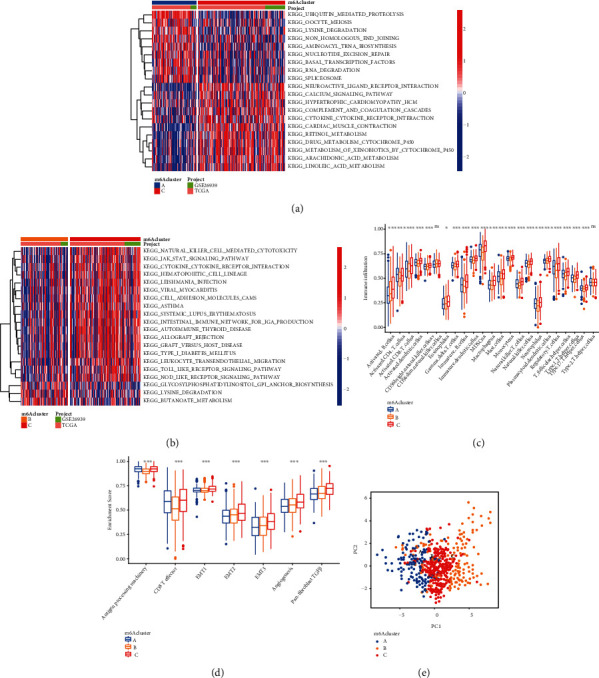
GSVA enrichment analysis showing the activation states of biological pathways in distinct m^6^A modification patterns. The heat map was used to visualize these biological processes, and red represented activated pathways, and blue represented inhibited pathways. (a) m^6^A cluster A vs. m^6^A cluster C; (b) m^6^A cluster B vs. m^6^A cluster C; (c) the abundance of each TME infiltrating cell in three m^6^A modification patterns. The upper and lower ends of the boxes represented the interquartile range of values. The lines in the boxes represented median value, and dots showed outliers. The asterisks represented the statistical *P* value (^∗^*P* < 0.05; ^∗∗^*P* < 0.01; ^∗∗∗^*P* < 0.001). (d) Differences in stroma-activated pathways including EMT, TGF beta, and angiogenesis pathways among three distinct m^6^A modification patterns. The statistical differences among the three modification patterns were tested by the one-way ANOVA test. The asterisks represented the statistical *P* value (^∗^*P* < 0.05; ^∗∗^*P* < 0.01; ^∗∗∗^*P* < 0.001). (e) Principal component analysis for the transcriptome profiles of three m^6^A modification patterns, showing a remarkable difference in transcriptome between different modification patterns.

To further understand the biological activity of these diverse m^6^A mutation patterns, we employed GSVA enrichment analysis (Figures 4(a) and 4(b)). From the results of GSVA analysis, we can see that A modification mode is mainly related to the tumor pathway, while C modification mode is strongly related to immune activation. Subsequently, the analysis of TME cell infiltration showed that m^6^A cluster C had abundant innate immune cell infiltration, including natural killer cells, macrophages, eosinophils, mast cells, MDSC, and plasma-like dendritic cells (Figure 4(c)). Analysis of related biological pathways showed that matrix activity in cluster C was significantly enhanced, such as transforming growth factor *β* (TGF*β*), epithelial-mesenchymal transition (EMT), and the activation of angiogenesis pathway (Figure 4(d)). To comprehensively evaluate the immunological characteristics of the methylation modification modes, we supplemented other validation methods. We examined the three modes using the ssGSEA method and 29 immune gene sets (Figure S5A). The tumor microenvironment features of these three subgroups were determined using the ESTIMATE findings. We discovered that cluster C had higher EstimateScore and StromalScore levels, while cluster A had lower levels of these scores (Wilcoxon test, *P* < 0.001) (Figure S5B). Cluster C also exhibited more HLA genes expressed, suggesting greater immunogenicity (Figure S5C). The three m^6^A alteration patterns displayed clear TME cell infiltration features, according to our findings. Cluster A is an immune desert phenotype characterized by immunosuppression, while cluster B is an immune inflammation phenotype characterized by moderate immune cell infiltration and immune activation, and cluster C is an immune exclusion phenotype characterized by innate immune cell infiltration and matrix activation. The expression of 23 m^6^A methylation moderators was studied using principal component analysis (PCA) to separate the patterns of three m^6^A methylation regulators (Figure 4(e)). The findings demonstrated that the three groups were differentiated, indicating that the transcription of 23 m^6^A methylation factors could identify the three categories.

### 3.4. The Creation of an m^6^A Methylation Score, as well as the Clinical and Transcriptome Aspects of m^6^A Methylation-Related Gene Clusters

**Figure 5:**
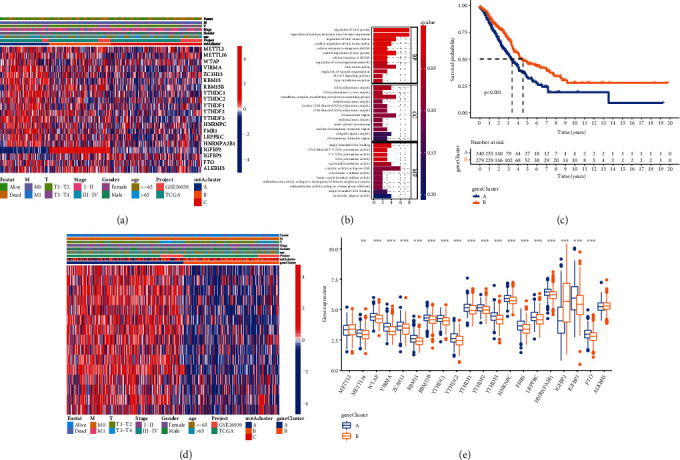
(a) Unsupervised clustering of 23 m^6^A regulators in the LUAD cohort. The m^6^A cluster, M, T, gender, age, stage, and survival status were used as patient annotations. Red represented a high expression of regulators, and blue represented low expression. (b) Functional annotation for m^6^A-related genes using GO enrichment analysis. The color depth of the barplots represented the number of genes enriched. (c) Kaplan-Meier curves indicated m^6^A modification genomic phenotypes were markedly related to the overall survival of patients in the LUAD cohort (*P* < 0.001, log-rank test). (d) Unsupervised clustering of 23 m^6^A regulators in the LUAD cohort. The gene cluster, m^6^A cluster, M, T, gender, age, stage, and survival status were used as patient annotations. Red represented a high expression of regulators, and blue represented low expression. (e) The expression of 23 m^6^A regulators in two gene clusters. The upper and lower ends of the boxes represented an interquartile range of values. The lines in the boxes represented median value, and black dots showed outliers. The asterisks represented the statistical *P* value (^∗^*P* < 0.05; ^∗∗^*P* < 0.01; ^∗∗∗^*P* < 0.001).

We studied the clinical data of 629 individuals to learn more about the characteristics of these changed phenotypes in terms of distinct clinical aspects and biological processes (Figure 5(a)). m^6^A clusters A, B, and C show different characteristics. Cluster A is highly expressed in most of the regulatory factors of m^6^A. However, in the regulatory factor IGFBP2, there are significant differences among the three subtypes. Cluster B has much greater levels of IGFBP2 expression than cluster A. Using the limma program, we assessed 68 phenotype1-related DEGs to better understand the probable biological activity of each m^6^A modification pattern (Figure S6). For DEG enrichment analysis, the clusterProfiler software suite was employed. These genes demonstrated a high enrichment of biological processes connected to m^6^A modification and virus, confirming that m^6^A alteration may play a key role in lung adenocarcinoma viral infection inflammation (Figure 5(b)). We used multivariate Cox survival analysis to select 15 genes closely related to survival from 68 m^6^A phenotype-related genes for unsupervised cluster analysis and divided the patients into different genomic subgroups to further validate this regulation mechanism (Figure S7 and Table S2). The unsupervised clustering algorithm also shows two different phenotypes of the m^6^A modified genome, which are consistent with the clustering of the m^6^A modified genome. The three groups were given the names m^6^A gene clusters A, B, and C (Figure S6). Two distinct gene clusters were discovered to have distinct distinctive genes, according to the research. A better prognosis is linked to cluster B (Figure 5(c)). The outlook for gene cluster A was not great. The two m^6^A gene clusters showed significant differential expression of m^6^A regulatory factors, which was consistent with the methylation modification process of m^6^A's predicted effects (Figure 5(e)).

### 3.5. Prognostic Value of m^6^A Related Phenotypes

**Figure 6:**
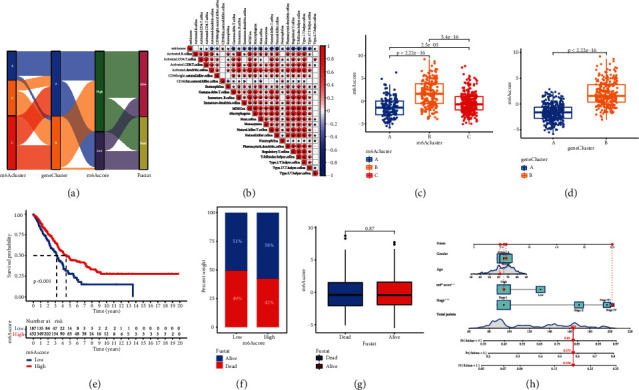
Construction of m^6^A signatures. (a) Alluvial diagram showing the changes of m^6^A clusters, gene cluster, m^6^A score, and survival status. (b) Correlations between m^6^A score and the known gene signatures in LUAD cohort using Spearman analysis. A negative correlation was marked with blue and a positive correlation with red. (c) Differences in m^6^A score among three m^6^A modification patterns in LUAD cohort (*P* < 0.001, Kruskal-Wallis test). (d) Differences in m^6^A score between two gene clusters in LUAD cohort. The Wilcoxon rank-sum test was used to compare the statistical difference between two gene clusters (*P* < 0.001). (e) Survival analyses for m^6^A score based on 629 patients with LUAD from two cohorts. (f) The proportion of survival status in low or high m^6^A score groups. Survival status: 51%/49% in the low m^6^A score groups and 58%/42% in the high m^6^A score groups. (g) Differences in m^6^A score between dead and alive groups (*P* = 0.87, Wilcoxon test). (h) m^6^A score related nomogram, the asterisks represented the statistical *P* value (^∗^*P* < 0.05; ^∗∗^*P* < 0.01; ^∗∗∗^*P* < 0.001).

Given the variability and complexity of m^6^A alteration, we developed the m^6^A score, a scoring system based on these phenotype-related genes, to assess the m^6^A modification pattern of lung cancer patients. The alluvial chart is used to display variations in the characteristics of specific patients (Figure 6(a)). We also investigate the link between known signature and m^6^A score to further show the properties of m^6^A signature (Figure 6(b)). The Kruskal-Wallis test revealed that the m^6^A scores of the m^6^A gene clusters differed significantly (Figures 6(c) and 6(d)). The median score for gene cluster A is the lowest, while the median score for gene cluster B is the greatest. Similarly, compared with other clusters, the m^6^A score of cluster A is significantly lower, while the score of m^6^A cluster B is significantly higher. Following that, we attempted to investigate the use of m^6^A score in predicting patient prognosis. Patients were split into high and low m^6^A score groups based on the appropriate cutoff value calculated by the survminer software tool. The survival benefit of patients with a higher m^6^A score was significant (Figures 6(e) and 6(f)). Then, patients were separated into alive or dead groups. The m^6^A score did not vary significantly between the two groups (Figure 6(g)). In addition, we also found that in young female patients, M0, stage I-II, and T3-4 patients, higher m^6^A score showed more significant survival advantage, which means that m^6^A score can also be used to access various clinical features of patients, such as age, gender, and clinical stage (Figure S8). The clinical data and m^6^A score were used to construct the m^6^A related nomogram, which confirmed that m^6^A score can be used to predict the outcome of LUAD (Figure 6(h)).

### 3.6. Characteristics of m^6^A Modification in Tumor Somatic Mutations

**Figure 7:**
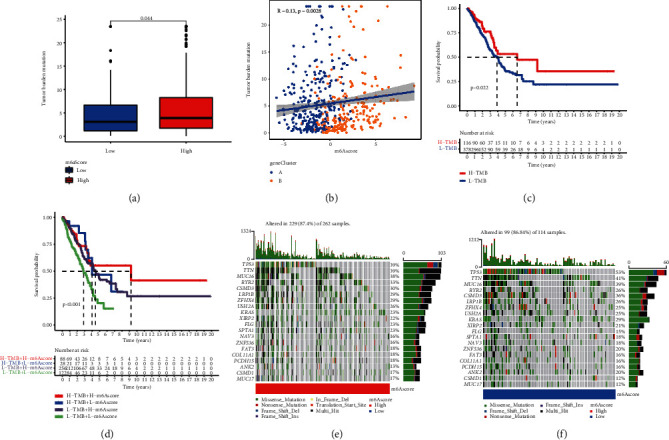
(a) Difference in tumor burden mutation between two m^6^A score groups in the LUAD cohort. The Wilcoxon rank-sum test was used to compare the statistical difference between two gene clusters (*P* = 0.044). (b) Correlations between m^6^A score and tumor burden mutation using Spearman analysis (*R* = 0.13, *P* = 0.0028). (c) Survival analysis of the high (*N* = 116) and low (*N* = 378) TMB groups in the LUAD cohort. (d) Survival analysis of distinct groups stratified by both TMB and m^6^A score (*P* < 0.022). (e, f) The waterfall plot of tumor somatic mutation established by those with high m^6^A score (e) and low m^6^A score (f). Each column represented individual patients. The upper barplot showed TMB; the number on the right indicated the mutation frequency in each gene. The right barplot showed the proportion of each variant type.

The link between the m^6^A score and tumor mutation burden (TMB) was then investigated (Figures 7(a) and 7(b)). The results suggest that the mutation load is positively correlated with the score of m^6^A, and the mutation load is higher in the group with a higher score of m^6^A. In particular, we investigated the effects of the characteristics of the m^6^Ascore and mutation load on survival (Figure 7(c)). We found that the survival advantage was more obvious in the high mutation load group. Another study combined mutation load and m^6^A score analysis showed that high mutation load and high m^6^A score group showed better survival advantage, while low mutation load and low m^6^A score group showed lower survival rate (Figure 7(d)). The map tools software program was used to examine the differences in somatic mutation distribution between upper and lower m^6^A score groups (Figures 7(e) and 7(f)). The group with a high m^6^A score had a higher mutation load than the group with a low m^6^A score. TMB was quantitatively analyzed, and it was shown that tumors with a higher m^6^A score had a higher TMB. In addition, m^6^A score and TMB had a strong positive connection. Individuals with a high TMB status showed a longer-lasting clinical response to anti-PD-1/PD-L1 immunotherapy, according to accumulating research. As a consequence, the foregoing findings imply that tumor m^6^A alteration patterns vary, which might be a critical role in determining clinical response to anti-PD-1/PD-L1 immunotherapy. Indirect evidence of m^6^A score's use in determining the outcome of immunotherapy has also been discovered.

### 3.7. Patterns of m^6^A Alteration in the Context of Anti-PD-1/L1 Immunotherapy

**Figure 8:**
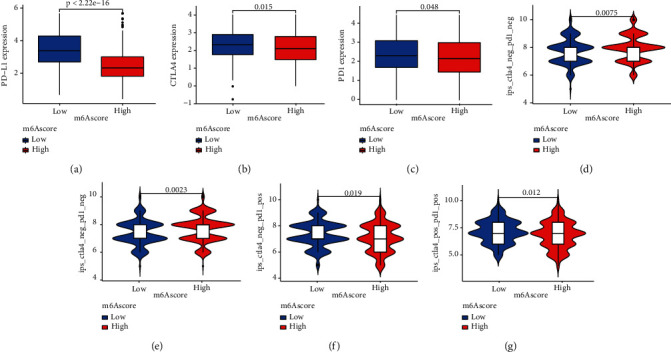
m^6^A modification patterns in the role of immunotherapy. (a) Differences in PD-L1 expression between low and high m^6^A score groups (*P* < 0.0001, Wilcoxon test). (b) Differences in CTLA4 expression between low and high m^6^A score groups (*P* = 0.015, Wilcoxon test). (c) Differences in PD1 expression between low and high m^6^A score groups (*P* = 0.048, Wilcoxon test). (d–g) Differences in immunotherapy effects between low and high m^6^A score groups (*P* < 0.05, Wilcoxon test).

In the treatment of a variety of tumor forms, immunotherapy has improved survival rates. It is crucial to figure out which patients will benefit the most. As a result, we investigated whether the m^6^A score might predict the success of immunotherapy by inhibiting four immunological checkpoints in the treatment group. The patients with low m^6^A score were significantly high in PD-L1, CTLA4, and PD1, which suggested that the patients may have a better response to immunotherapy (Figures 8(a)–8(c)). We further observed the response of the m^6^A score group to immunotherapy and found that the treatment effect of the low score group was better in patients with PD1 positive (Figures 8(d)–8(g)).

### 3.8. Inhibition of NOL10 Suppresses LC Cell Proliferation and Migration

**Figure 9:**
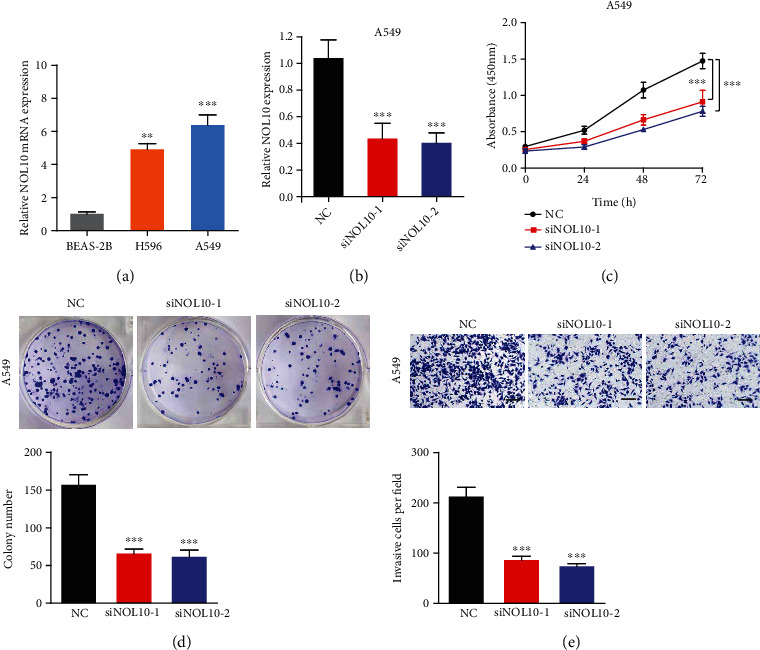
Inhibition of NOL10 suppresses LC cell proliferation and migration of lung cancer cells in vitro. (a) Relative expression of NOL10 in three cell lines. (b) qRT-PCR to detect the relative silencing levels of NOL10 in the A549 cell line. (c) The CCK-8 assay was applied to detect the efficiency of NOL10 knockdown on the proliferation of the A549 cell line. (d) Images of the colony formation assay after knockdown of NOL10 in A549 cell line. (e) Images of the transwell assay results after knockdown of NOL10 in A549 cell line.

The expression of NOL10 in lung cancer (LC) cell lines (H596 and A549), normal lung epithelial cells (BEAS-2B), and LC was examined to further confirm the function of NOL10. H596 and A549 cells have considerably greater NOL10 expression than BEAS-2B cells (Figure 9(a)). Then, using si-NOL10 transfection, we were able to effectively knock down NOL10 expression in A549 cells (Figure 9(b)). Lower expression of NOL10 substantially decreased the capacity of A549 cells to proliferate, according to the findings of CCK-8 and colony formation assays (Figures 9(c) and 9(d)). The transwell experiment revealed that suppressing NOL10 expression with siRNA reduced the number of invading cells significantly (Figure 9(e)). These findings suggest that knocking down NOL10 inhibits LC cell growth and migration.

## 4. Discussion

Due to the interplay of several m^6^A regulatory variables, m^6^A alteration may have a significant role in the occurrence, progression, and treatment of a range of illnesses [26]. Because most current research focuses on a single TME cell type or a single regulatory factor, the overall profile of TME infiltration mediated by several m^6^A regulatory elements has not been adequately addressed. Clarifying the role of different m^6^A modification patterns in TME cell invasion will help us better understand the antitumor immune response of TME and contribute to the development of more efficient immunotherapy medicines in the clinic.

According to 23 regulatory factors of m^6^A, this study showed three different methylation patterns of m^6^A. These three patterns have different characteristics of TME cell infiltration. Immunosuppression characterizes group A, adaptive immune activation characterizes group B, corresponding to the immunological inflammatory phenotype, and innate immunity and matrix activation characterize group C, indicating immunological exclusion, relating to the immunological desert phenotype. Noninflammatory cancers include immune exclusion and immunological depletion characteristics [31, 32]. Despite the presence of a large number of immune cells, the immune exclusion phenotype kept immune cells in the stroma around the tumor cell nest instead of allowing them to penetrate the parenchyma. Stroma may either stay inside the tumor capsule or invade the tumor and create immune cells that seem to be within the tumor. Immune cells are present in tumors with an immune exclusion phenotype, but they stay in the stroma surrounding the tumor cell nest instead of reaching the tumor parenchyma, according to a previous study [33, 34]. Immunological tolerance, immunological ignorance, and a shortage of activation and activated T cells are all linked to the immune desert phenotype [35]. It validated the accuracy of our immunophenotypic categorization of various m^6^A alteration patterns when combined with the invasion characteristics of TME cells in each cluster. It is not unexpected, however, that group C had active immunity but a bad prognosis, based on the features of TME cell infiltration generated by various m^6^A alteration modalities. Group C is the so-called “hot tumor” [36]. There are many immune cells in this kind of tumor, but they do not play a role. The effect of immune checkpoint inhibition therapy for this kind of tumor will be better [37].

In this study, it has been confirmed that the differences of mRNA transcriptome between different m^6^A modification patterns are significantly related to biological pathways. We believe that these differentially expressed genes are characteristic genes related to m^6^A. According to the characteristic genes of m^6^A, we determined the optimal classification method and divided the characteristic genome into two subtypes. The two subtypes are also mainly related to viruses and immunity. This also confirmed that the modification of m^6^A is of great significance for TME. As a result, a thorough examination of the m^6^A alteration pattern will help us better comprehend the invasion properties of TME cells. Given the individual variability of m^6^A alteration, quantifying the alteration pattern of a particular tumor is critical. We created the m^6^A gene signature scoring method to assess the pattern of m^6^A change in LUAD patients. Immunological exclusion phenotype m^6^A modification models scored lower, whereas immune-inflammatory phenotype m^6^A modification models scored better. m^6^A score was discovered to be an independent biomarker for the prediction of LUAD after a thorough investigation. This demonstrates that the m^6^A score may be used to look at a single tumor's m^6^A modification pattern to predict the TME or tumor immunophenotype invasion pattern.

The findings also reveal that there is a substantial positive association between the m^6^A score and the burden of tumor mutation. Patients with higher somatic cell TMB are related to improved response, long-term survival, and long-term therapeutic advantages when undergoing immune checkpoint blocking medication, according to clinical trials and preclinical investigations [6, 38]. Individually changed genes may regulate resistance or susceptibility to immunotherapy [39–41]. For clearly altered genes in LUAD, such as TTN and USH2A, the mutant's m^6^A score was much lower than that of the wild type; however, there was no discernible difference between wild type and mutant in MUC16 and USH2A. These findings will open up new areas of research into the mechanism of m^6^A methylation in tumor somatic mutation, the development of TME characteristics, and the impact of immune checkpoint inhibitors.

Finally, the m^6^A score may be used to analyze the methylation patterns of m^6^A thoroughly. The corresponding score further defines the microenvironment, and immune characteristics of individual patients determine the immune phenotype of the tumor and guide more effective treatment management. We also discovered that the m^6^A score may be utilized to assess patients' clinical features, such as tumor inflammation stage and tumor mutation load. Similarly, m^6^A score may be utilized as a stand-alone prognostic biomarker to predict patient survival. Furthermore, we verified the expression of m^6^A related enzymes and the role hub gene closely related to survival in LC cell lines. We discovered that suppressing the expression of the hub gene NOL10 greatly slowed lung cancer cell growth and migration. Our findings suggest new ways to improve patient's clinical responses to immunotherapy, such as identifying diverse tumor immunophenotypes and advocating for personalized tumor immunotherapy in the future.

## Data Availability

Publicly available datasets were used in this study. These data can be found in the Gene Expression Omnibus (GEO) and in the Cancer Genome Atlas (TCGA) database.

## Supplementary Materials

Supplementary 1 Figure S1 The protein levels of RBMX, METTL16, and LRPPRC in lung cancer cells and normal lung epithelial cell BEAS-2B. ∗*P* <0.05, ∗∗*P* <0.01, and ∗∗∗*P* <0.001.

Supplementary 2 Figure S2 (A) The prognostic analyses for 23 m^6^A regulators in the LUAC cohorts using a univariate Cox regression model. Hazard ratio>1 represented risk factors for survival, and hazard ratio<1 represented protective factors for survival. (B) The prognostic analyses for DEGs using a univariate Cox regression model. Hazard ratio>1 represented risk factors for survival, and hazard ratio<1 represented protective factors for survival.

Supplementary 3 Figure S3 Consensus matrices of the LUAC cohort for *k* = 2, 4, 5.

Supplementary 4 Figure S4 (A-D) Consensus matrices of the LUAD cohort of GSE30219 and GSE37745 datasets for *k* = 3. (E) Survival analyses for the three m^6^A modification patterns in LUAD cohort using Kaplan-Meier curves including 167 cases in m^6^A cluster A, 137 cases in m^6^A cluster B, and 181 cases in m^6^A cluster C. The m^6^A cluster B showed significantly better overall survival than the other two m^6^A cluster (*P* = 0.022, log-rank test).

Supplementary 5 Figure S5 Immunophenotype of LUAD patients with three m^6^A stratification: (A) comprehensive evaluation of immunological characteristics by the ssGSEA algorithm; (B) comparison of EstimateScore and StromalScore among the three modes; (C) comparison of HLA gene expression level among the three modes. ∗*P* <0.05, ∗∗*P* <0.01, and ∗∗∗*P* <0.001.

Supplementary 6 Figure S6 (A) 68 m^6^A phenotype-related genes shown in Venn diagram. (B) Consensus matrices of the LUAC cohort.

Supplementary 7 Figure S7 15 survival-related DEGs were included in the PPI network. The nodes indicated proteins. The edges represented proteins' interaction.

Supplementary 8 Figure S8 Survival analyses for patients with m^6^A score groups in the age, gender, M, stage, and T.

Supplementary 9 Table S1 The prognostic analyses for 23 m^6^A regulators in the LUAC cohorts using a univariate Cox regression model.

Supplementary 10 Table S2 The prognostic analyses for DEGs using a univariate Cox regression model.

## References

[1] Richards T. B., Henley S. J., Puckett M. C. (2017). Lung cancer survival in the United States by race and stage (2001-2009): findings from the CONCORD-2 study. *Cancer*.

[2] Bray F., Ferlay J., Soerjomataram I., Siegel R. L., Torre L. A., Jemal A. (2018). Global cancer statistics 2018: GLOBOCAN estimates of incidence and mortality worldwide for 36 cancers in 185 countries. *CA: a Cancer Journal for Clinicians*.

[3] Chen Z., Fillmore C. M., Hammerman P. S., Kim C. F., Wong K. K. (2014). Non-small-cell lung cancers: a heterogeneous set of diseases. *Nature Reviews. Cancer*.

[4] Aran D., Hu Z., Butte A. J. (2017). xCell: digitally portraying the tissue cellular heterogeneity landscape. *Genome Biology*.

[5] Travis W. D., Brambilla E., Nicholson A. G. (2015). The 2015 World Health Organization classification of lung tumors: impact of genetic, clinical and radiologic advances since the 2004 classification. *Journal of Thoracic Oncology*.

[6] Lievense L. A., Sterman D. H., Cornelissen R., Aerts J. G. (2017). Checkpoint blockade in lung cancer and mesothelioma. *American Journal of Respiratory and Critical Care Medicine*.

[7] McNutt M. (2013). Cancer immunotherapy. *Science*.

[8] Pitt J. M., Marabelle A., Eggermont A., Soria J. C., Kroemer G., Zitvogel L. (2016). Targeting the tumor microenvironment: removing obstruction to anticancer immune responses and immunotherapy. *Annals of Oncology*.

[9] Quail D. F., Joyce J. A. (2013). Microenvironmental regulation of tumor progression and metastasis. *Nature Medicine*.

[10] Fridman W. H., Pagès F., Sautès-Fridman C., Galon J. (2012). The immune contexture in human tumours: impact on clinical outcome. *Nature Reviews. Cancer*.

[11] Fridman W. H., Zitvogel L., Sautès–Fridman C., Kroemer G. (2017). The immune contexture in cancer prognosis and treatment. *Nature Reviews. Clinical Oncology*.

[12] Jansen C. S., Prokhnevska N., Kissick H. T. (2019). The requirement for immune infiltration and organization in the tumor microenvironment for successful immunotherapy in prostate cancer. *Urologic Oncology*.

[13] Zhao B. S., Roundtree I. A., He C. (2017). Post-transcriptional gene regulation by mRNA modifications. *Nature Reviews. Molecular Cell Biology*.

[14] Patil D. P., Chen C. K., Pickering B. F. (2016). m^6^A RNA methylation promotes _XIST_ -mediated transcriptional repression. *Nature*.

[15] Alarcón C. R., Lee H., Goodarzi H., Halberg N., Tavazoie S. F. (2015). *N*
^6^-methyladenosine marks primary microRNAs for processing. *Nature*.

[16] Boccaletto P., Machnicka M. A., Purta E. (2018). MODOMICS: a database of RNA modification pathways. 2017 update. *Nucleic Acids Research*.

[17] Yang Y., Hsu P. J., Chen Y. S., Yang Y. G. (2018). Dynamic transcriptomic m^6^A decoration: writers, erasers, readers and functions in RNA metabolism. *Cell Research*.

[18] Li F., Wang H., Huang H., Zhang L., Wang D., Wan Y. (2020). m6A RNA methylation regulators participate in the malignant progression and have clinical prognostic value in lung adenocarcinoma. *Frontiers in Genetics*.

[19] Zhang Y., Liu X., Liu L., Li J., Hu Q., Sun R. (2020). Expression and prognostic significance of m6A-related genes in lung adenocarcinoma. *Medical Science Monitor*.

[20] Colaprico A., Silva T. C., Olsen C. (2016). TCGAbiolinks: an R/Bioconductor package for integrative analysis of TCGA data. *Nucleic Acids Research*.

[21] Hartigan J. A., Wong M. A. (1979). Algorithm AS 136: a K-means clustering algorithm. *Journal of the Royal Statistical Society. Series C (Applied Statistics)*.

[22] Wilkerson M. D., Hayes D. N. (2010). ConsensusClusterPlus: a class discovery tool with confidence assessments and item tracking. *Bioinformatics*.

[23] Chao Y., Shang J., Ji W. (2020). ALKBH5-m^6^A-FOXM1 signaling axis promotes proliferation and invasion of lung adenocarcinoma cells under intermittent hypoxia. *Biochemical and Biophysical Research Communications*.

[24] Yang L., Li J., Fu S. (2019). Up-regulation of insulin-like growth factor binding protein-3 is associated with brain metastasis in lung adenocarcinoma. *Molecules and Cells*.

[25] Yu H., Zhang Z. (2021). ALKBH5-mediated m6A demethylation of lncRNA RMRP plays an oncogenic role in lung adenocarcinoma. *Mammalian Genome*.

[26] Zhang X., Zhang S., Yan X. (2021). m6A regulator-mediated RNA methylation modification patterns are involved in immune microenvironment regulation of periodontitis. *Journal of Cellular and Molecular Medicine*.

[27] Ritchie M. E., Phipson B., Wu D. (2015). Limma powers differential expression analyses for RNA-sequencing and microarray studies. *Nucleic Acids Research*.

[28] Zeng D., Li M., Zhou R. (2019). Tumor microenvironment characterization in gastric cancer identifies prognostic and immunotherapeutically relevant gene signatures. *Cancer Immunology Research*.

[29] Sotiriou C., Wirapati P., Loi S. (2006). Gene expression profiling in breast cancer: understanding the molecular basis of histologic grade to improve prognosis. *Journal of the National Cancer Institute*.

[30] Hazra A., Gogtay N. (2016). Biostatistics series module 3: comparing groups: numerical variables. *Indian Journal of Dermatology*.

[31] Chen D. S., Mellman I. (2017). Elements of cancer immunity and the cancer-immune set point. *Nature*.

[32] Qian C., Cao X. (2018). Dendritic cells in the regulation of immunity and inflammation. *Seminars in Immunology*.

[33] Turley S. J., Cremasco V., Astarita J. L. (2015). Immunological hallmarks of stromal cells in the tumour microenvironment. *Nature Reviews. Immunology*.

[34] Gajewski T. F., Woo S. R., Zha Y. (2013). Cancer immunotherapy strategies based on overcoming barriers within the tumor microenvironment. *Current Opinion in Immunology*.

[35] Joyce J. A., Fearon D. T. (2015). T cell exclusion, immune privilege, and the tumor microenvironment. *Science*.

[36] Galon J., Bruni D. (2019). Approaches to treat immune hot, altered and cold tumours with combination immunotherapies. *Nature Reviews. Drug Discovery*.

[37] Duan Q., Zhang H., Zheng J., Zhang L. (2020). Turning cold into hot: firing up the tumor microenvironment. *Trends in Cancer*.

[38] Tsai H. F., Hsu P. N. (2017). Cancer immunotherapy by targeting immune checkpoints: mechanism of T cell dysfunction in cancer immunity and new therapeutic targets. *Journal of Biomedical Science*.

[39] de Azevedo R. A., Shoshan E., Whang S. (2020). MIF inhibition as a strategy for overcoming resistance to immune checkpoint blockade therapy in melanoma. *Oncoimmunology*.

[40] Georganaki M., van Hooren L., Dimberg A. (2018). Vascular targeting to increase the efficiency of immune checkpoint blockade in cancer. *Frontiers in Immunology*.

[41] Correale P., Saladino R. E., Giannarelli D. (2020). HLA expression correlates to the risk of immune checkpoint inhibitor-induced pneumonitis. *Cell*.

