# Supplementary figures and images for: Methylation Pattern Mediated by m6A Regulator and Tumor Microenvironment Invasion in Lung Adenocarcinoma
Source: Oxid Med Cell Longev. 2022 Jan 5;2022:2930310. doi: 10.1155/2022/2930310 (PMC8756160; doi:10.1155/2022/2930310)

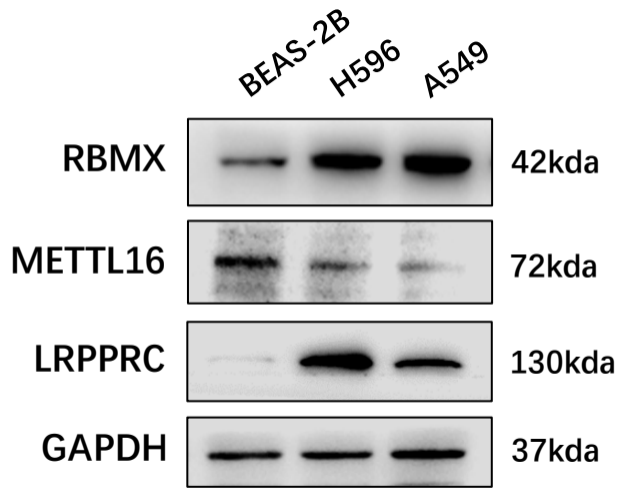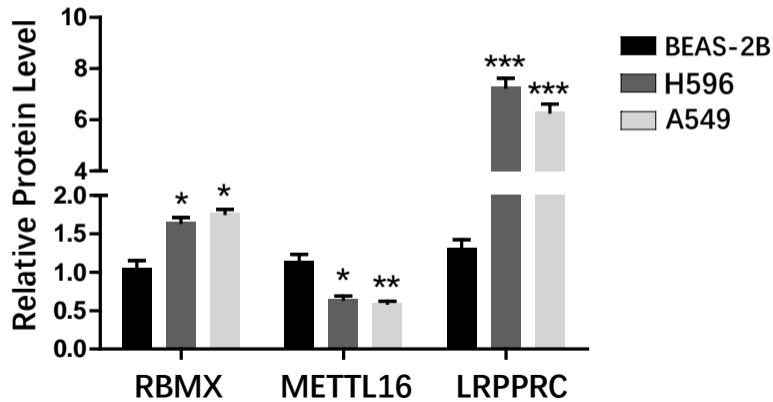

Supplement: Supplementary 1 — Figure S1 The protein levels of RBMX, METTL16, and LRPPRC in lung cancer cells and normal lung epithelial cell BEAS-2B. ∗P <0.05, ∗∗P <0.01, and ∗∗∗P <0.001. [file 2930310.f1.pdf]

A

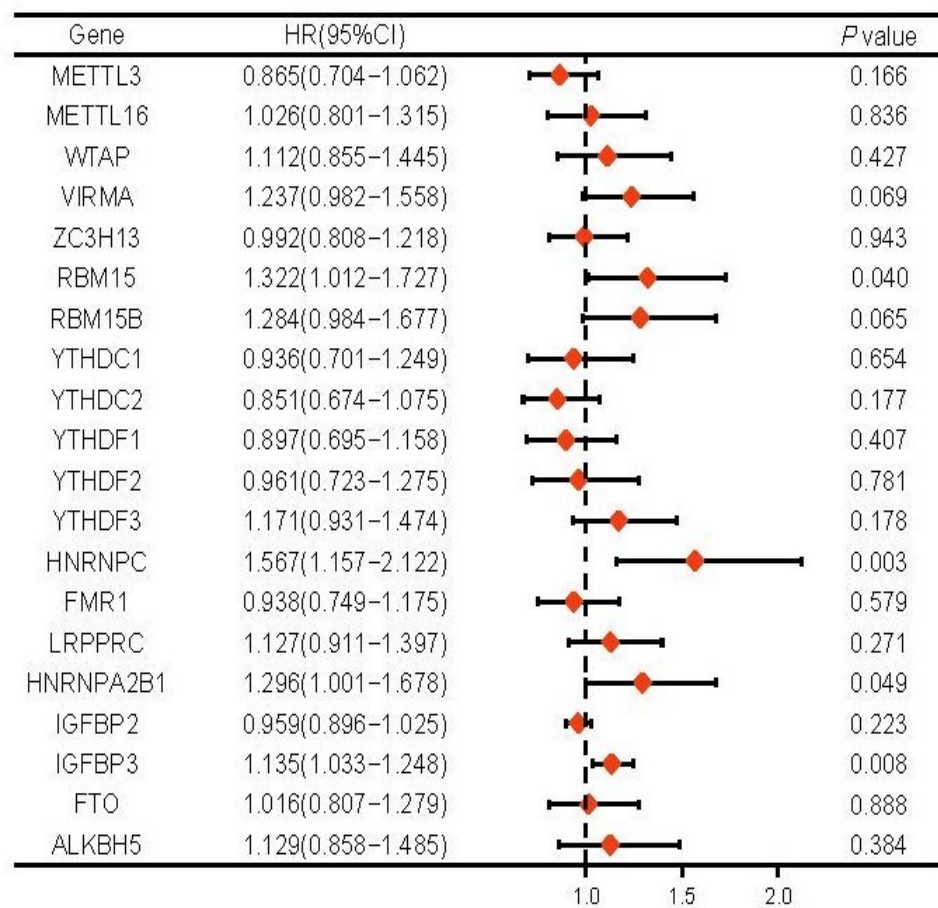

B

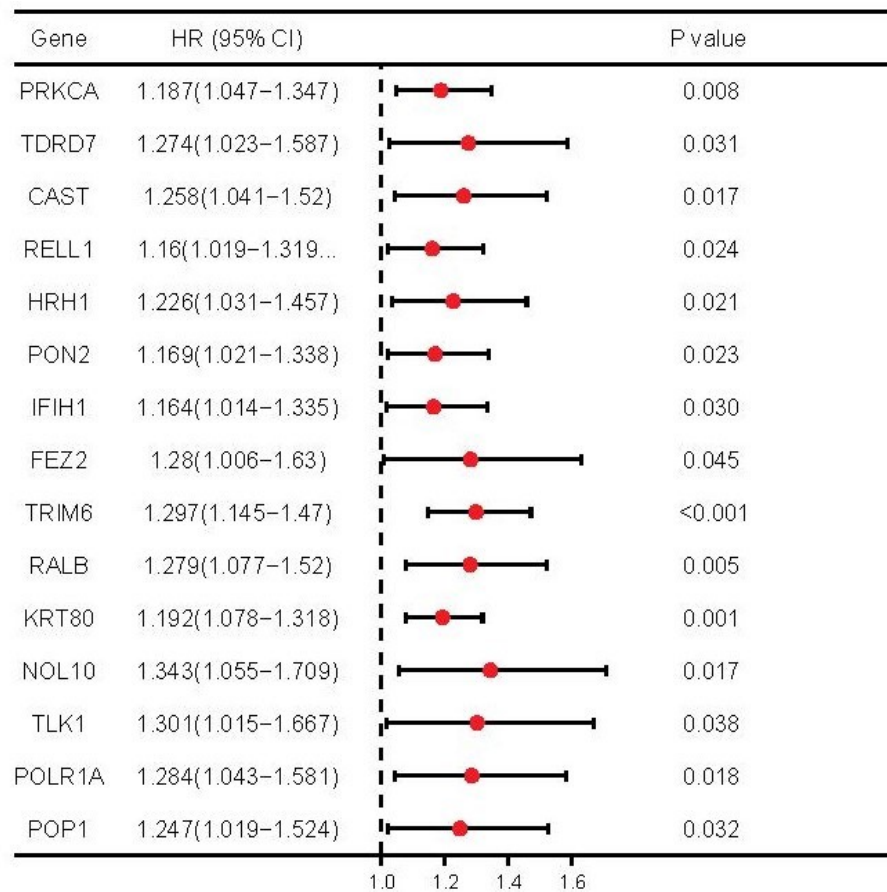

Supplement: Supplementary 2 — Figure S2 (A) The prognostic analyses for 23 m6A regulators in the LUAC cohorts using a univariate Cox regression model. Hazard ratio>1 represented risk factors for survival, and hazard ratio<1 represented protective factors for survival. (B) The prognostic analyses for DEGs using a univariate Cox regression model. Hazard ratio>1 represented risk factors for survival, and hazard ratio<1 represented protective factors for survival. [file 2930310.f2.pdf]

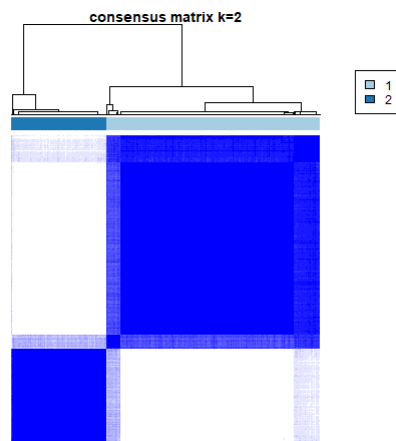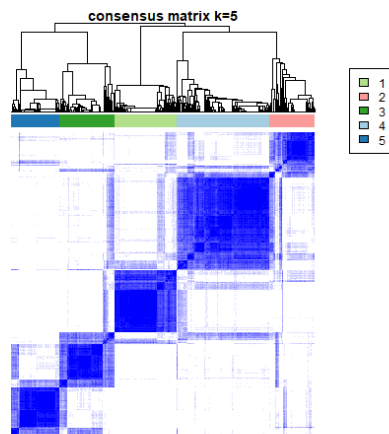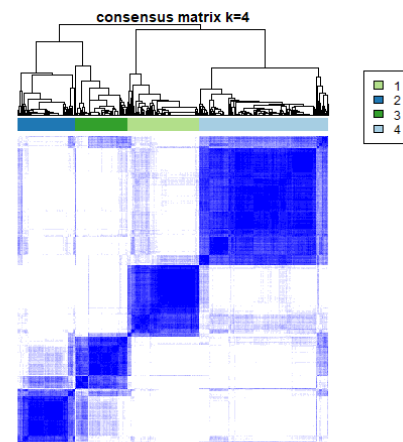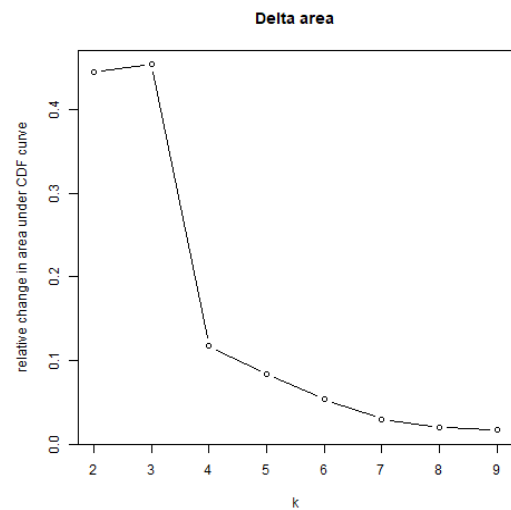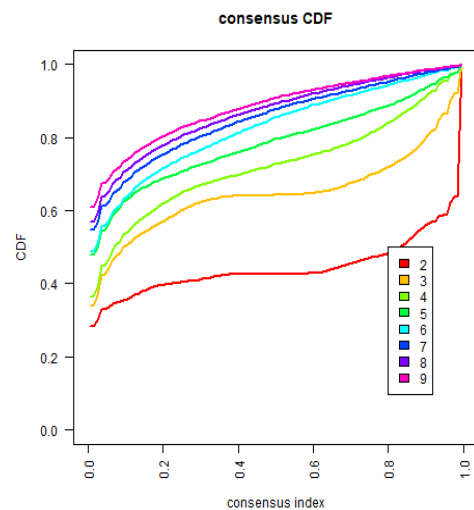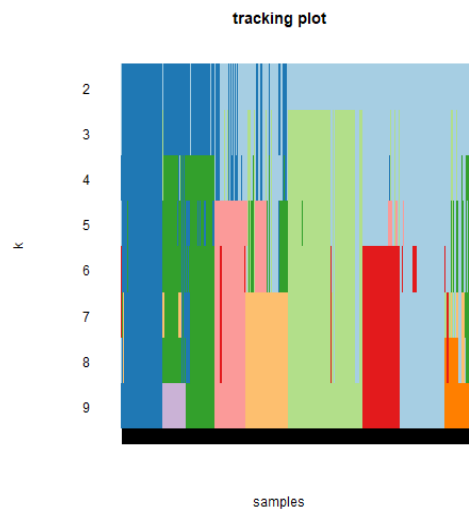

Supplement: Supplementary 3 — Figure S3 Consensus matrices of the LUAC cohort for k = 2, 4, 5. [file 2930310.f3.pdf]

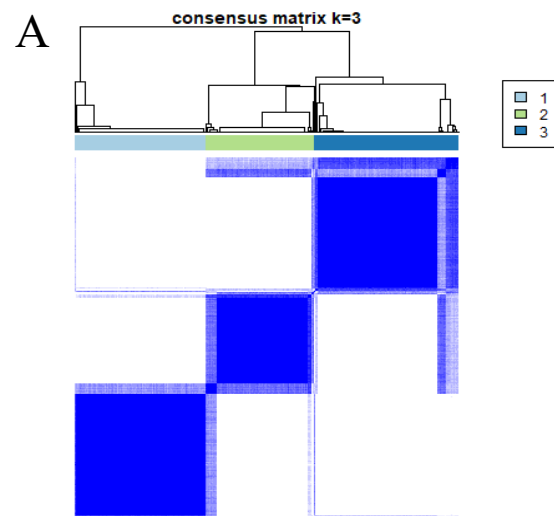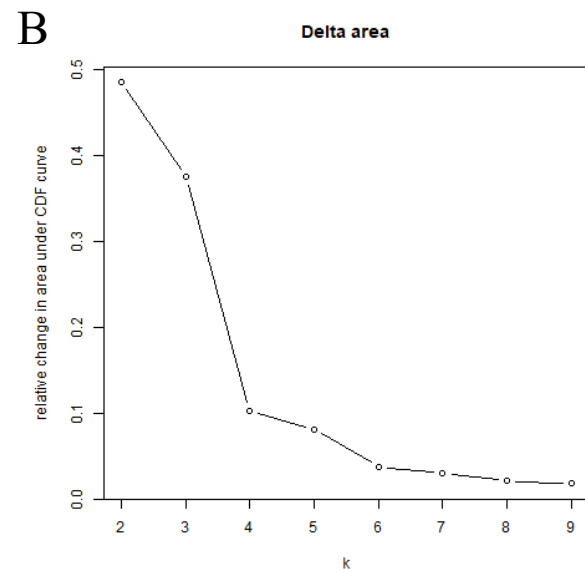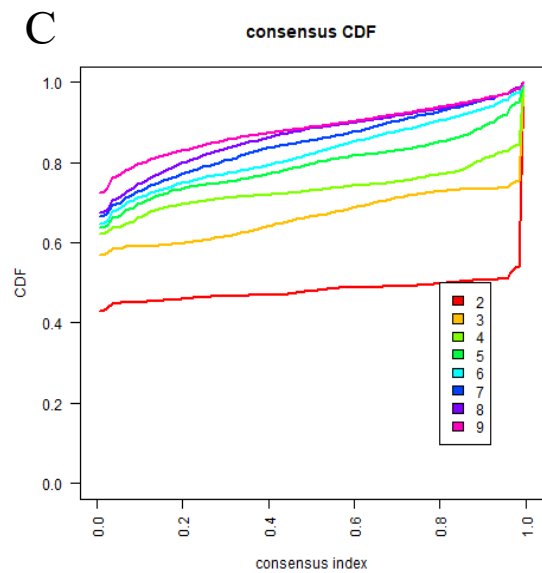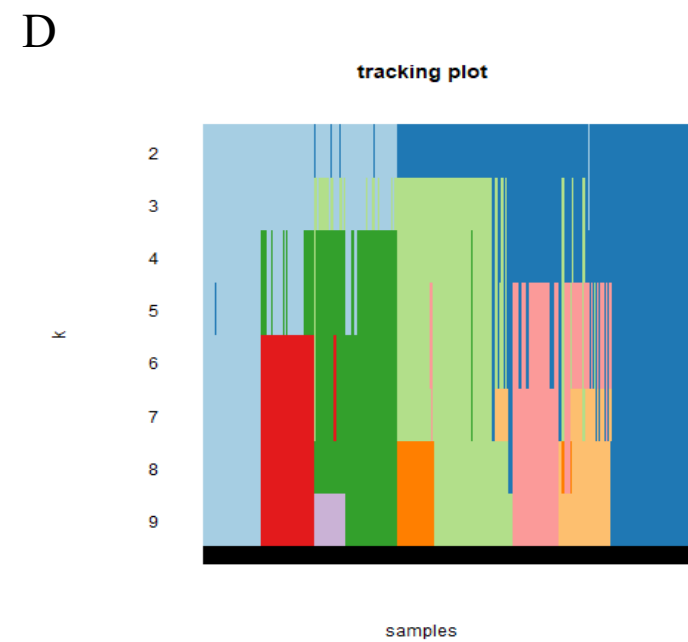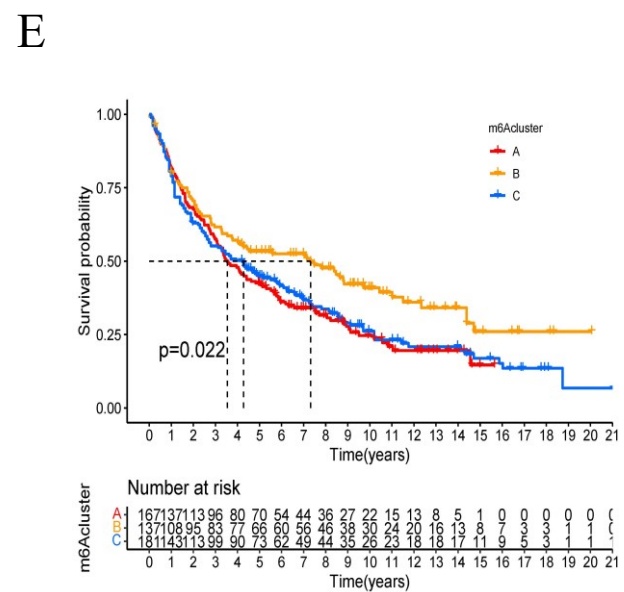

Supplement: Supplementary 4 — Figure S4 (A-D) Consensus matrices of the LUAD cohort of GSE30219 and GSE37745 datasets for k = 3. (E) Survival analyses for the three m6A modification patterns in LUAD cohort using Kaplan-Meier curves including 167 cases in m6A cluster A, 137 cases in m6A cluster B, and 181 cases in m6A cluster C. The m6A cluster B showed significantly better overall survival than the other two m6A cluster (P = 0.022, log-rank test). [file 2930310.f4.pdf]

A

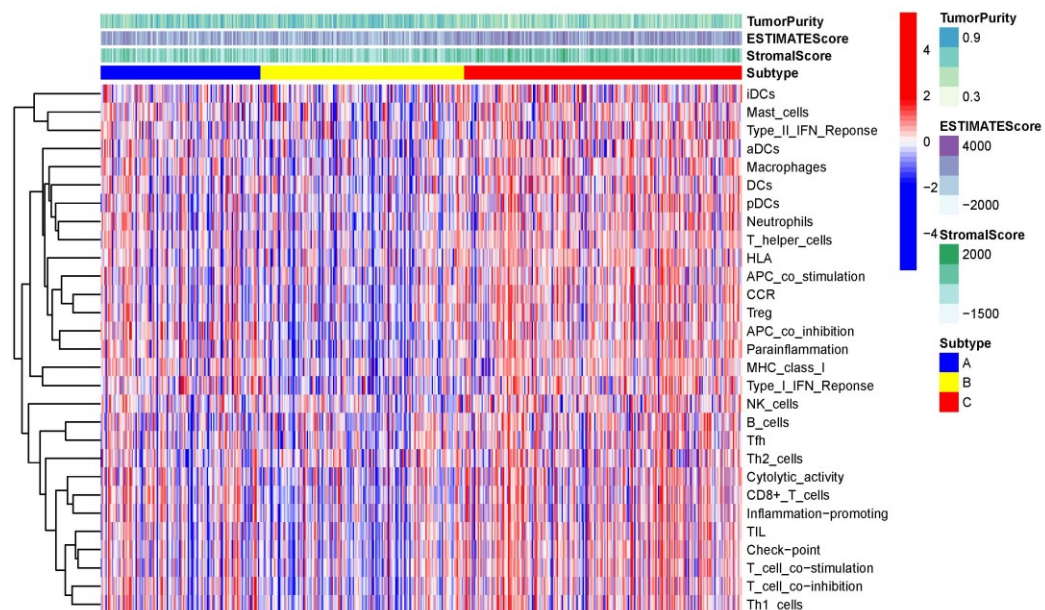

B

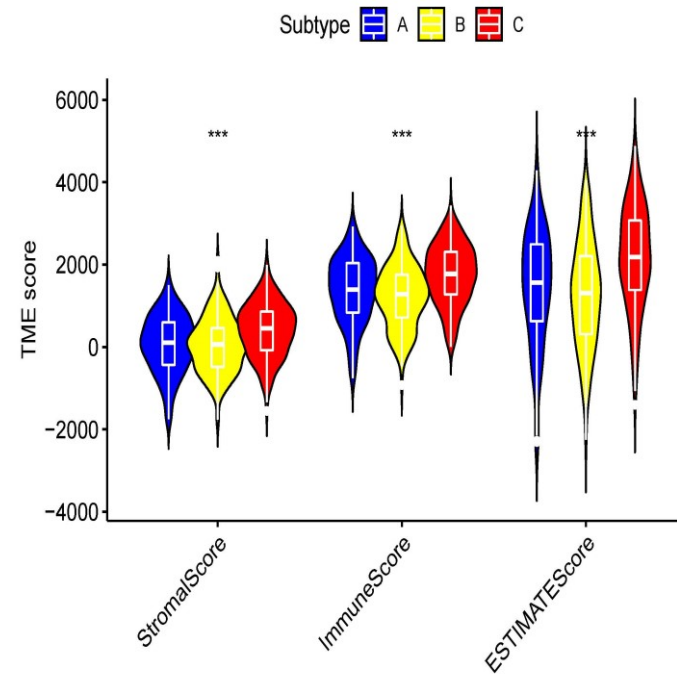

C

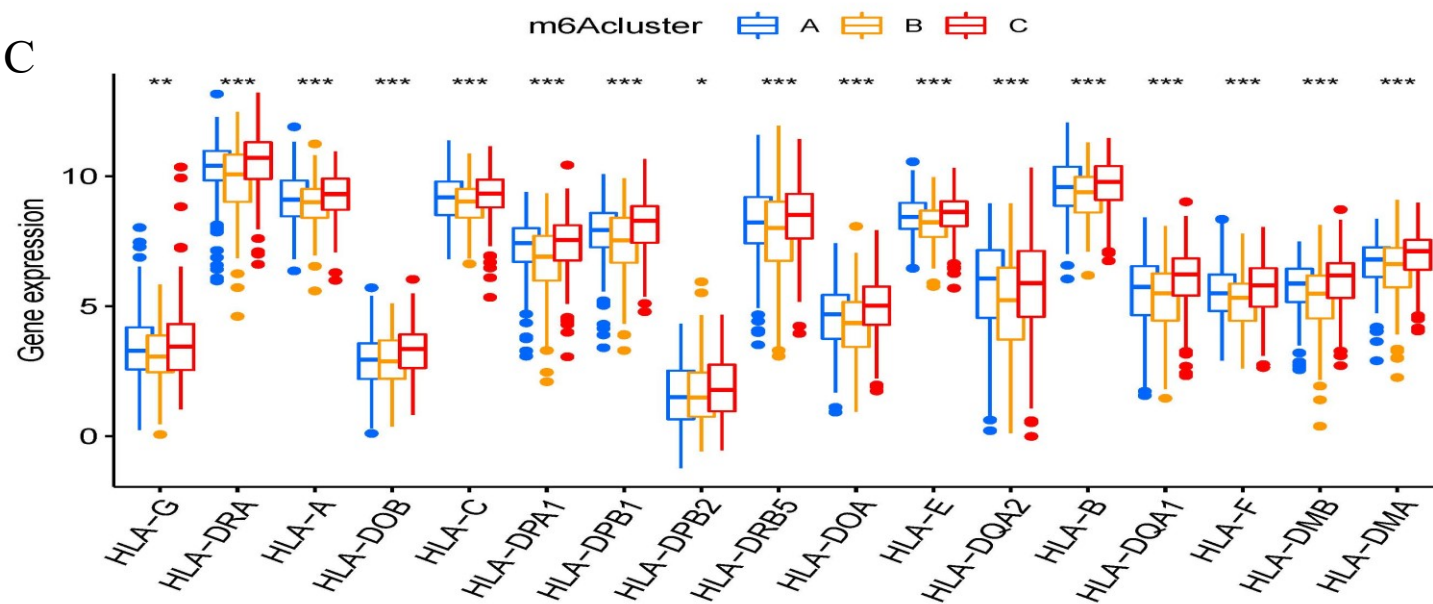

Supplement: Supplementary 5 — Figure S5 Immunophenotype of LUAD patients with three m6A stratification: (A) comprehensive evaluation of immunological characteristics by the ssGSEA algorithm; (B) comparison of EstimateScore and StromalScore among the three modes; (C) comparison of HLA gene expression level among the three modes. ∗P <0.05, ∗∗P <0.01, and ∗∗∗P <0.001. [file 2930310.f5.pdf]

A

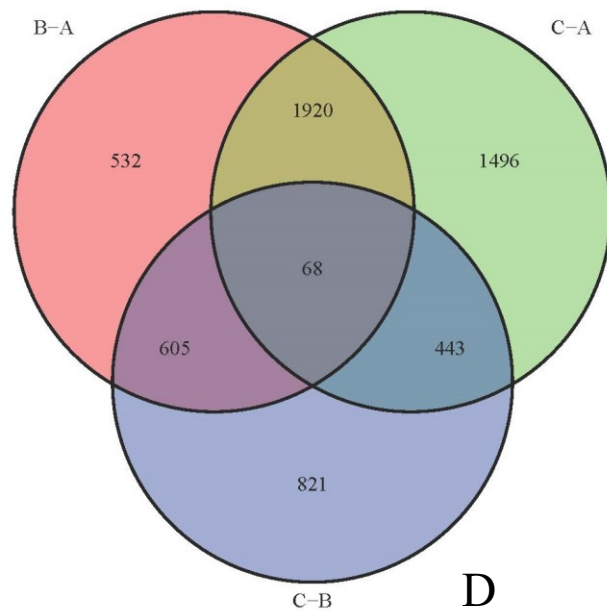

B

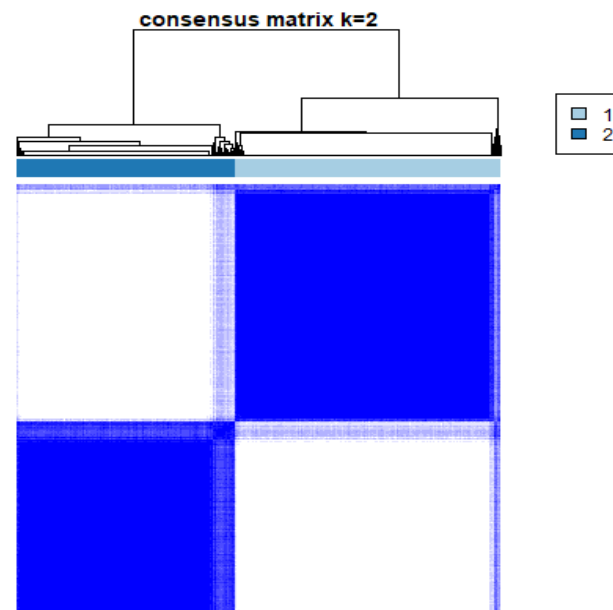

C

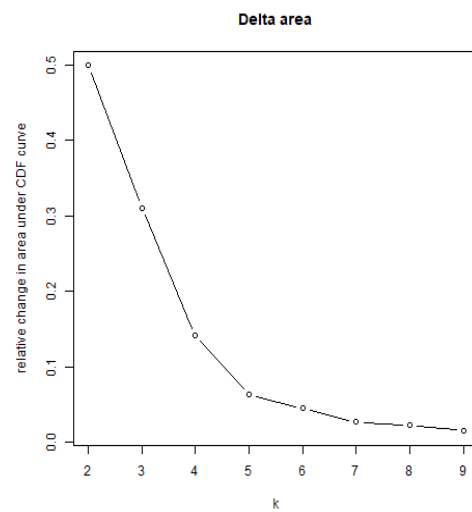

D

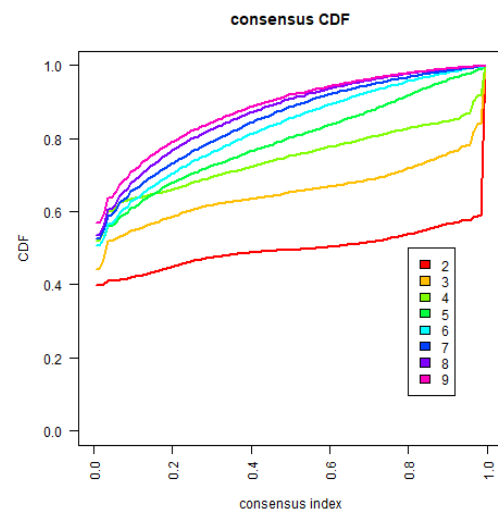

E

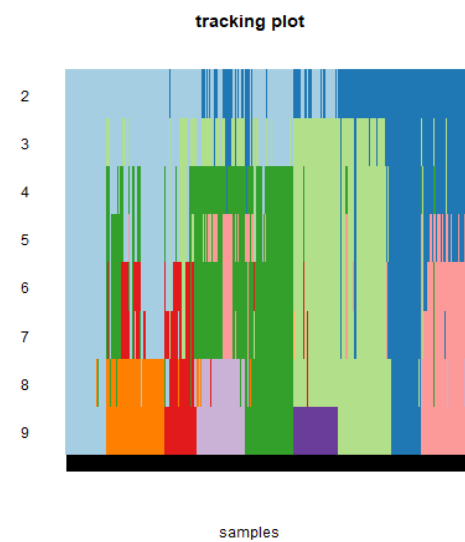

Supplement: Supplementary 6 — Figure S6 (A) 68 m6A phenotype-related genes shown in Venn diagram. (B) Consensus matrices of the LUAC cohort. [file 2930310.f6.pdf]

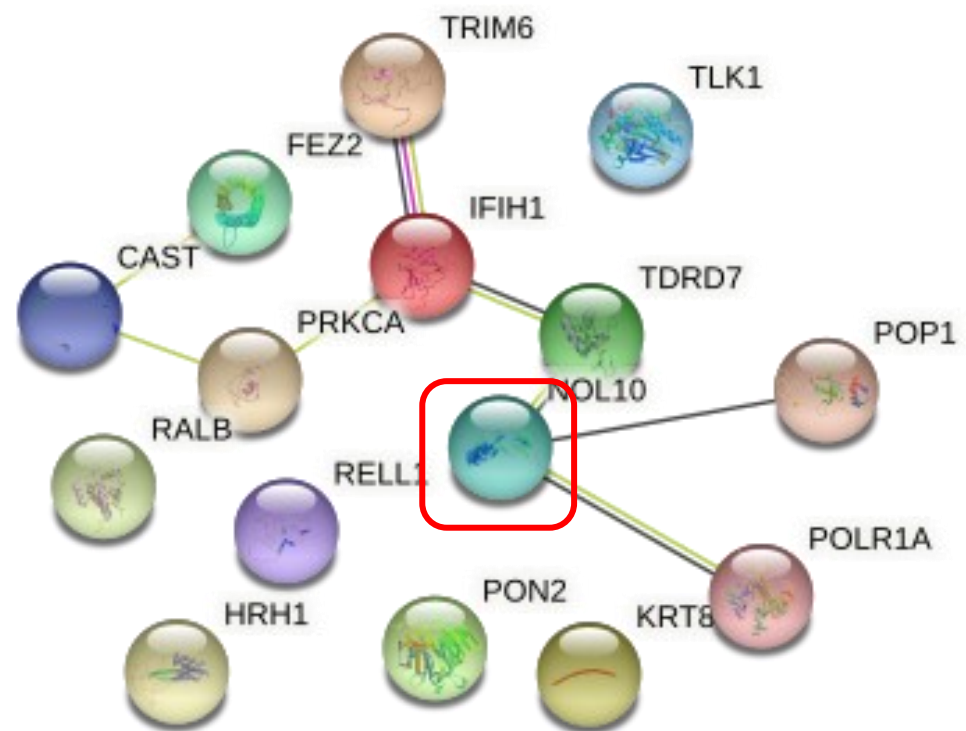

Supplement: Supplementary 7 — Figure S7 15 survival-related DEGs were included in the PPI network. The nodes indicated proteins. The edges represented proteins' interaction. [file 2930310.f7.pdf]

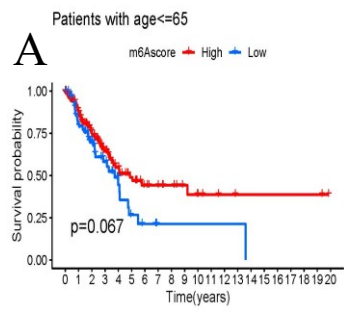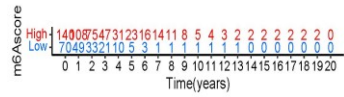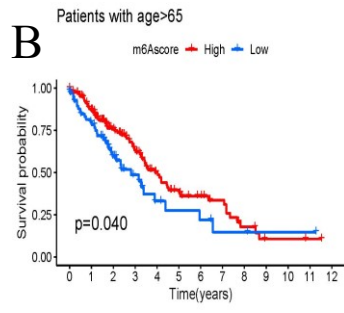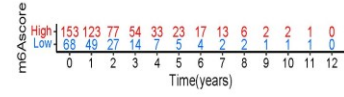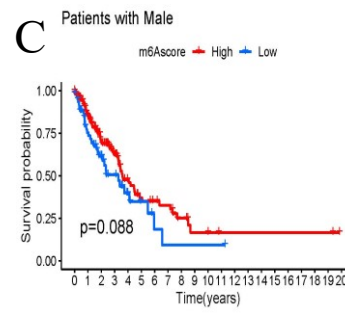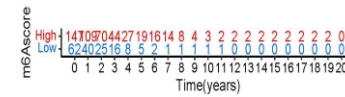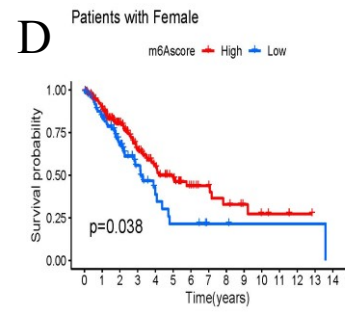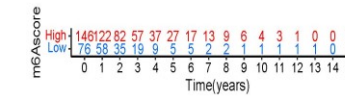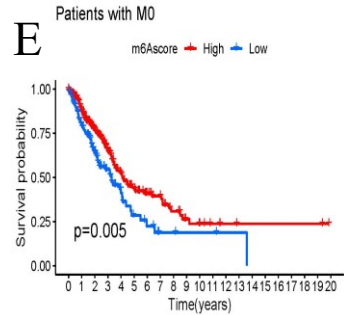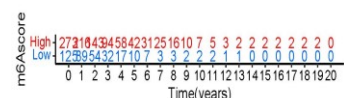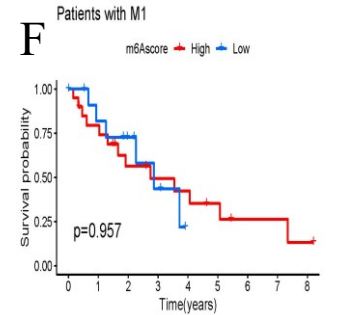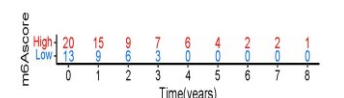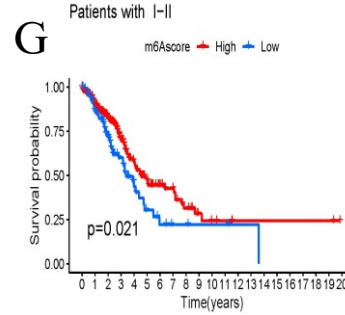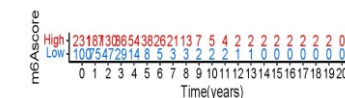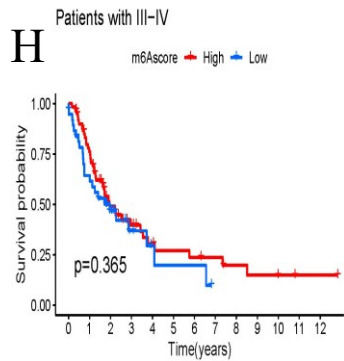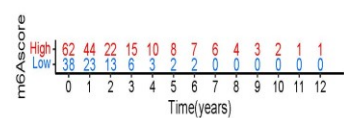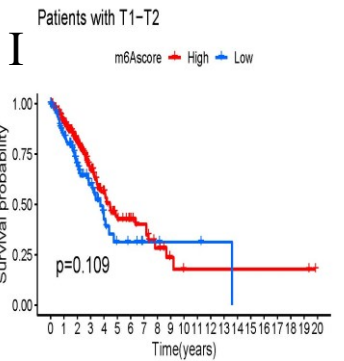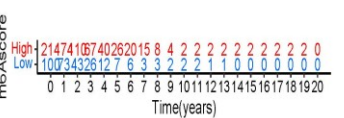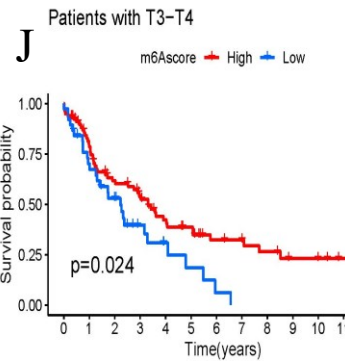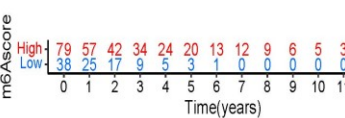

Supplement: Supplementary 8 — Figure S8 Survival analyses for patients with m6A score groups in the age, gender, M, stage, and T. [file 2930310.f8.pdf]
